# Obstructive Sleep Apnea After Supracricoid Laryngeal Surgery (OPHL II): A Monocentric Prospective Pilot Study

**DOI:** 10.3390/cancers18081212

**Published:** 2026-04-10

**Authors:** Massimo Mesolella, Salvatore Allosso, Fabio Perrotta, Carlo Iadevaia, Carmela Cirillo, Nicola Serra, Pasquale Capriglione, Martina Ricciardiello, Anna Leoni, Anna Rita Fetoni

**Affiliations:** 1 Unit of Otorhinolaryngology, Department of Neuroscience, Reproductive Sciences and Dentistry, University of Naples Federico II, 80131 Naples, Italy; massimo.mesolella@unina.it (M.M.); pasquale.capriglione@unina.it (P.C.); martinaricciardielo@icloud.com (M.R.); annarita.fetoni@unina.it (A.R.F.); 2 Department of Translational Medical Sciences, University of Campania “L. Vanvitelli”, 80131 Naples, Italy; fabio.perrotta@unicampania.it (F.P.); dott.carlo.iadevaia@gmail.com (C.I.); carmela.cirillo1990@gmail.com (C.C.); leonianna4@gmail.com (A.L.); 3 Unit of Respiratory Medicine “Luigi Vanvitelli”, A.O. dei Colli, Monaldi Hospital, 80131 Naples, Italy; 4 Department of Neuroscience, Reproductive Sciences and Dentistry, Audiology Section, University of Naples Federico II, Via Pansini 5, 80131 Naples, Italy; 5 Hearing and Balance Unit, Department of Head and Neck, Federico II University Hospital, Via Pansini 5, 80131 Naples, Italy

**Keywords:** OPHL, laryngeal cancer, supracricoid laryngectomy, OSA, polysomnography, AHI

## Abstract

**Background/Objectives**: Obstructive sleep apnea (OSA) is increasingly observed in patients undergoing supracricoid laryngeal surgery; however, the impact of postoperative anatomical changes on sleep-disordered breathing remains insufficiently characterized. This pilot study aimed to assess the incidence and severity of OSA after Open Partial Horizontal Laryngectomy type II (OPHL II) and to evaluate correlations between polysomnographic parameters and radiologic measurements of the neolarynx. **Methods**: A prospective observational cohort study was conducted on ten patients who underwent OPHL II between 2019 and 2024 and were evaluated at least one year postoperatively. The sample size was determined using a conservative estimate appropriate for a pilot prospective study, which required a long postoperative follow-up period of at least one year. All patients completed Stop-Bang, Berlin, and Epworth questionnaires and underwent overnight polysomnography. Cervical CT scans were used to measure airway length to the vocal cords (ALVC), supralaryngeal tract horizontal (SVTH) and vertical (SVTV) segments, and the base-of-tongue–to–cervical-body distance (BTCB). **Results**: OSA was detected in all patients: 40% mild, 30% moderate, and 30% severe. Mean AHI was 25.5 ± 18.9 events/h, and OSA severity strongly correlated with AHI (rho = 0.94; *p* < 0.0001). Among radiologic parameters, SVTV showed a positive correlation with OSA severity (rho = 0.82; *p* = 0.0035), while BTCB demonstrated a significant negative correlation (rho = −0.71; *p* = 0.0207). No significant associations were found for ALVC or SVTH. **Conclusions**: Supracricoid laryngectomy produces anatomical changes that predispose patients to OSA. Radiologic metrics—particularly SVTV and BTCB—appear to be meaningful predictors of OSA severity. A multidisciplinary approach is essential for early diagnosis and management. Due to the small number of patients enrolled larger multicenter studies are needed to confirm these findings and define radiologic criteria associated with postoperative OSA.

## Simple Summary

Patients who undergo supracricoid laryngeal surgery for laryngeal cancer often experience important changes in the anatomy of the upper airway. These changes can make breathing during sleep more difficult and may lead to obstructive sleep apnea, a disorder characterized by repeated pauses in breathing and drops in oxygen levels. In this study, we evaluated patients one year after surgery to understand how often sleep apnea occurs and which anatomical changes are most closely related to its severity. By combining sleep studies with radiologic measurements, we identified specific structural changes—particularly a longer vertical airway tract and a reduced space behind the tongue—that were strongly associated with more severe disease. These findings highlight the importance of early evaluation and collaboration between ear, nose, and throat specialists, pulmonologists, and radiologists to ensure timely diagnosis and treatment.

## 1. Introduction

Obstructive sleep apnea (OSA) is a complex, multifactorial disorder characterized by partial or complete obstruction of the upper airway, often caused by hypopharyngeal collapse [1]. Sleep irregularities, characterized by respiratory interruptions with episodes of desaturation and micro-arousals, lead to daytime sleepiness and, above all, to a long-term, increased in the incidence of cardiovascular disease [2,3,4].

The prevalence of OSA in the general population is between 2% and 4%, and is directly related to age [3,4].

General risk factors include gender, age, obesity, diabetes, and smoking. The incidence increases until the age of 60, with a gender difference (male > female). Local factors that can predispose to OSA include: macroglossia, tonsillar hypertrophy, and craniofacial characteristics, including posterior mandibular position [1,5,6].

OSA is diagnosed through polysomnography (PSG). According to the American Academy of Sleep Medicine (AASM), the severity of OSA can be classified based on the apnea/hypopnea index (AHI): severe for AHI ≥ 30, moderate for AHI < 30 and ≥15, and mild for AHI < 15 and ≥5 [3,7,8].

Generally, the anatomical sites responsible for determining the onset of OSA are represented by the velopendulum, tongue, and hypopharynx. The only laryngeal subsite taken into consideration is the epiglottis. Despite the limited description in the literature, clinical experience also suggests an increase in OSA incidence in patients undergoing laryngeal-conserving surgery, particularly supracricoid laryngectomy [9].

Laryngeal cancer is the most common ENT tumor in the head and neck area [10]. Laryngeal conservative surgery is performed to ensure disease eradication while preserving swallowing, speaking, and breathing through the natural airway [11,12,13,14].

Regarding conservative surgery, we distinguish between type I Horizontal Open Partial Laryngectomy (OPHL I), which involves the removal of the supraglottic portion of the larynx, and type II Horizontal Open Partial Laryngectomy (OPHL II), which involves the removal of the entire supracricoid portion of the larynx, with or without sparing the epiglottis. It is possible to spare a cricoarytenoid unit to ensure functional outcomes in terms of speaking and swallowing [15,16].

If the epiglottis is spared, the reconstructive surgical technique involves cricohyoidoepiglottopexy (CHEP); if the epiglottis is sacrificed, the procedure is called cricohyoidopexy (CHP) [17].

Anatomical alterations of the neolarynx resulting from conservative surgery often cause the onset of OSA. Teixeira et al. [18] compared patients undergoing vertical and horizontal laryngectomy, finding an increase in OSA in those undergoing vertical surgery (mean 36.9 vs. 11.1). Decotte et al. [19] investigated postoperative complications responsible for respiratory problems in patients undergoing partial laryngeal surgery (postoperative edema, arytenoid mucosal flap). Rombaux et al. [20], Israel et al. [21] reported their experience with the onset of OSA after laryngeal reconstructive surgery.

The aim of this study was to investigate whether anatomical alterations of the neolarynx following conservative surgery are linked to the onset of OSA.

## 2. Materials and Methods

We conducted a prospective pilot study at the ENT Unit of Federico II University Hospital to evaluate the onset of OSA in patients undergoing supracricoid reconstructive laryngeal surgery (OPHL type II) for laryngeal cancer by correlating anatomical changes detected by CT scans with the onset of OSA.

We considered patients who underwent OPHL type II surgery between 2019 and 2024 and were evaluated for OSA at least one year after surgery. All patients were informed regarding the methods, aims, and scope of the study.

The inclusion criteria were as follows: (1) histologically confirmed squamous cell carcinoma of the larynx; (2) indication from the Multidisciplinary Team for reconstructive surgical treatment according to the Head and Neck Cancers NCC clinical practice guidelines [22]; (3) informed consent.

The exclusion criteria were as follows: (1) patients who had received adjuvant or neoadjuvant therapy (chemotherapy and/or radiotherapy) to the head and neck area; (2) patients who had not undergone decannulation, (3) patients with dysphagia, (4) patients with type 1 diabetes mellitus, (5) patients with synchronous or metastatic tumors; (6) incomplete data records; (7) less than one year of follow-up without evidence of disease, (8) craniofacial anomalies (retrognathia, micrognathia, high-arched palate, and congenital malformations).

The surgical procedures were always performed by the same experienced surgical team.

A total of 10 patients were enrolled, all of whom were briefed on the procedures to be performed and provided written informed consent forms.

Patients were categorized according to the type of surgery, sex, and age, and on risk factors indicative of OSA: number of cigarettes smoked before surgery, BMI, hypertension, dyslipidemia, type II diabetes mellitus, cardiac rhythm abnormalities, and history of chronic coronary syndrome (CAD).

Each patient completed the Stop-Bang, Berlin, and Epworth Scale assessment questionnaires (All. A). The questionnaires were administered by the same medical team while the patient was at rest, ten minutes apart.

The Stop-Bang Questionnaire consists of 3 categories related to the risk of having obstructive sleep apnea (OSA): low risk; intermediate risk; high risk.

The Berlin Questionnaire consists of 3 categories related to the risk of having sleep apnea.

The Epworth Sleepiness Scale assesses a patient’s likelihood of falling asleep in certain situations, as opposed to feeling tired, using a scale from 0 to 3 (low-high probability).

All patients underwent an ENT examination with rhinofibrolaryngoscopy to assess the Friedman and Mallampati scales [23].

The Friedman classification is used to evaluate the anatomy of the oro-hypopharynx, considering the normal position of the tongue, and allows us to divide patients into five classes (I, IIa, IIb, III, and IV).

The Mallampati classification is used to evaluate the visibility of the pharyngeal structures, considering the protruding tongue, and allows us to divide patients into four classes (I, II, III, and IV).

Rhinofibrolaryngoscopy revealed no anatomical alterations, such as arytenoid or neoglottis edema, that could affect the outcome one year after surgery.

Each patient underwent overnight polysomnography using the VitalPlus Night^®^ instrument (VitalAire Italia SpA—Via del Bosco Rinnovato 9, 200030 Milanofiori Nord—Milan, Italy). For each patient, the Apnea Hypopnea Index (AHI) was assessed according to the criteria of the American Academy of Sleep Medicine; the number of total apneas, central apneas, and mixed apneas, the number of hypopneas, the time below 90 (T90) which corresponds to the percentage of time in which oxygen saturation is less than 90 percent, the Oxygen Desaturation Index (ODI) which corresponds to the number of desaturations greater than or equal to 3–4% compared to baseline, the number of percent desaturations, and the mean percent oxygen saturation.

The polysomnographic evaluation, from preparation to interpretation of the results, was performed by two pneumologists with experience in the field of sleep disorders from the University of Campania—“L. Vanvitelli”.

**Figure 1:**
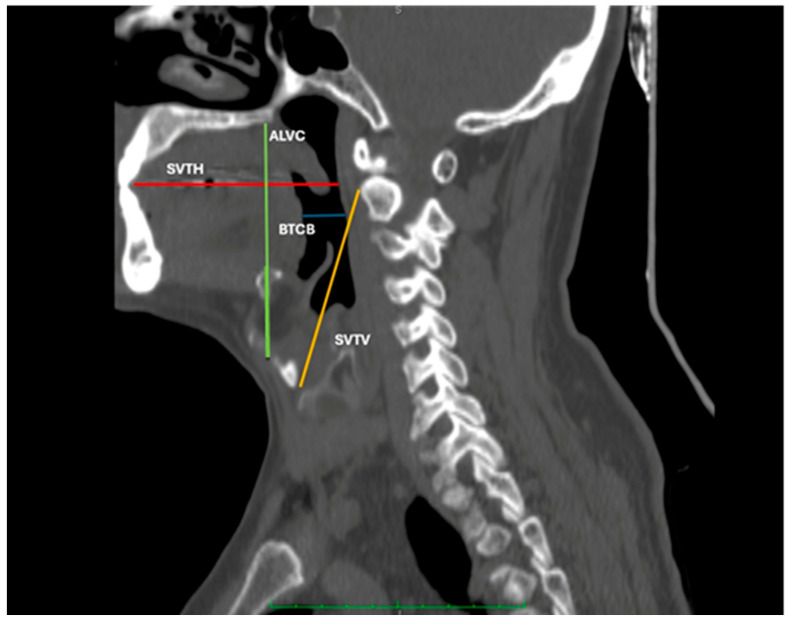
Radiological parameters.

Subsequently, the patients underwent CT scans Somatom GoTop^®^ 64 slices (Siemens Healthcare S.r.l—Via Vipiteno, 4, 20128 Milano—Italy) of the face and neck to evaluate several parameters:

Airway length to vocal cord (ALVC) that indicates the distance between the posterior edge of hard palate and the glottic plane; horizontal segment of supralaryngeal vocal cords (SVTH) that indicates the distance between incisor teeth and second cervical body; vertical segment of supralaryngeal vocal cord tract (SVTV) that indicates the distance between second cervical body and glottic plane; base of the tongue to cervical body (BTCB) that indicates the distance between the base of the tongue and the cervical body (Figure 1). Radiological values were evaluated blindly by the same radiologist with respect to the polysomnography results. The CT images were obtained with the same instrument (therefore, the same number of cuts for each patient). This allowed for standardized measurements. The radiologist measured the parameters in centimeters using the “ruler” function in the company’s DICOM file viewing program.

This study was conducted in accordance with relevant guidelines and regulations. It was approved by the Institutional Review Board Committee of the Federico II University of Naples, Naples, Italy (2022/207472).

### 2.1. Primary Outcome Criterion

The primary outcome of this study was OSA severity. OSA severity was assigned to a 3-point ordinal score based on standard polysomnographic criteria:

1 = mild,
2 = moderate,
3 = severe.

### 2.2. Primary Explanatory Variables

The primary explanatory variables of the study were the radiological parameters of the neolarynx, each representing a specific postoperative anatomical alteration. Four measurements were obtained from postoperative cross-sectional imaging:

1. ALVC (Airwaves Length to Vocal Cord): represents vertical supraglottic expansion or narrowing. It was measured in centimeters on sagittal CT slices at the level of the reconstructed ventricular cavity.
2. BTCB (Base of the Tongue to Cervical Body): reflects anteroposterior shortening or elongation of the reconstructed airway. It was measured as the linear anteroposterior distance between the base of the tongue and the anterior margin of the second cervical bone on sagittal CT reconstruction.
3. SVTH (horizontal segment of supralaryngeal vocal cord tract): quantifies horizontal thickening or reduction in supraglottic tissues.It was assessed as the anteroposterior distance of the supraglottic segment on sagittal CT reconstruction, measuring the distance between the incisors and the second cervical bone.
4. SVTV (vertical segment of supralaryngeal vocal cord tract): indicates volumetric enlargement or reduction in the supraglottic airway. It was calculated as diameter transverse between the second cervical bone and the glottic plane on sagittal CT reconstruction and expressed as a total airway volume.

These radiological parameters characterize measurable anatomical alterations of the neolarynx after supracricoid laryngectomy and were used to evaluate their impact on OSA severity, which served as the primary outcome.

### 2.3. Study Hypothesis

We hypothesized that greater postoperative anatomical alterations—quantified by variations in ALVC, BTCB, SVTH, and SVTV—would be associated with higher OSA severity scores, considering the Likert 3-point ordinal scale.

### 2.4. Sample Size Estimate

To investigate the relationship between anatomical alterations of the neolarynx following conservative surgery and the onset of OSA, we estimate for this pilot study the minimum sample size to ensure that the preliminary analysis would have sufficient precision to detect clinically meaningful trends, despite the exploratory nature of the investigation.

The sample size was defined using Cochran’s formula. The formula is shown below,

$$N=\frac{z^{2}\pi \left(1-\pi \right)}{\varepsilon^{2}}$$

where *π* represents the hypothesized prevalence of the impact of modified radiological parameter measurements in patients who underwent supracricoid reconstructive laryngeal surgery on obstructive sleep apnea, and ε denotes the accepted error in the evaluation of the sample size. In detail, we considered a 95% z-score, an error margin of *ε* = 35%, and a hypothesized prevalence of correct diagnosis of *π* = 50%. Since no prior data on this topic were available, we assumed maximum variability (*π* = 50%). We also adopted a relatively large margin of error (*ε* = 35%) because this is a pilot study. Consequently, we hypothesized that the estimated prevalence of the impact of radiological parameters on OSA could range between 15% and 85%.

Based on the above considerations, the minimum estimated sample size was 8 patients consecutively enrolled according to the selected inclusion and exclusion criteria. Finally, to reduce statistical biases due to information/data loss caused by the long study period and possible unexpected events, the sample size was increased to 10 patients.

Finally, we emphasize that this sample size estimation was intended to support an exploratory pilot study and to provide preliminary insights, rather than to ensure adequate statistical power for definitive hypothesis testing.

### 2.5. Statistical Analysis

Data were presented as number and percentage for categorical variables, and continuous data were expressed as the mean and standard deviation (SD), or median and interquartile range (IQR = [Q1, Q3]).

The Shapiro–Wilk test was used to determine if a variable was normally distributed. The Spearman correlation coefficient rho was computed to evaluate the degree of association between two variables when the distribution of the variables was not normal. OSA severity was assigned a 3-point ordinal score: mild = 1, moderate = 2, severe = 3.

Finally, all tests with *p*-value (*p*) < 0.05 were considered significant. The statistical analysis was performed using the Matrix Laboratory (MATLAB) analytical toolbox version 2008 (MathWorks, Natick, MA, USA). Running on a 32-bits Windows operating system.

## 3. Results

The statistical analyses were performed on sample of 10 consecutive patients composed of 80% males and 20% females, with ages ranging from 55 to 75 years, with a mean age of 64.8 years and standard deviation of 6.6. In Table 1, the characteristics including risk factors and some parameters linked to apneas were reported.

In Table 2 we reported two sections. The first section refers to the self-assessment questionnaires scores for our sample, including the obstructive sleep apnea (OSA) evaluation in patients for at least one year after OPHL type II surgery. The second section is related to sleep quality parameters such as Apnea Hypopnea Index (AHI), Oxygen Desaturation Index (ODI), time below 90% saturation (T90%), and the number of events below 90% saturation (N90%).

In Table 3 we reported the correlation analysis between OSA with radiological and sleep quality parameters.

**Figure 2:**
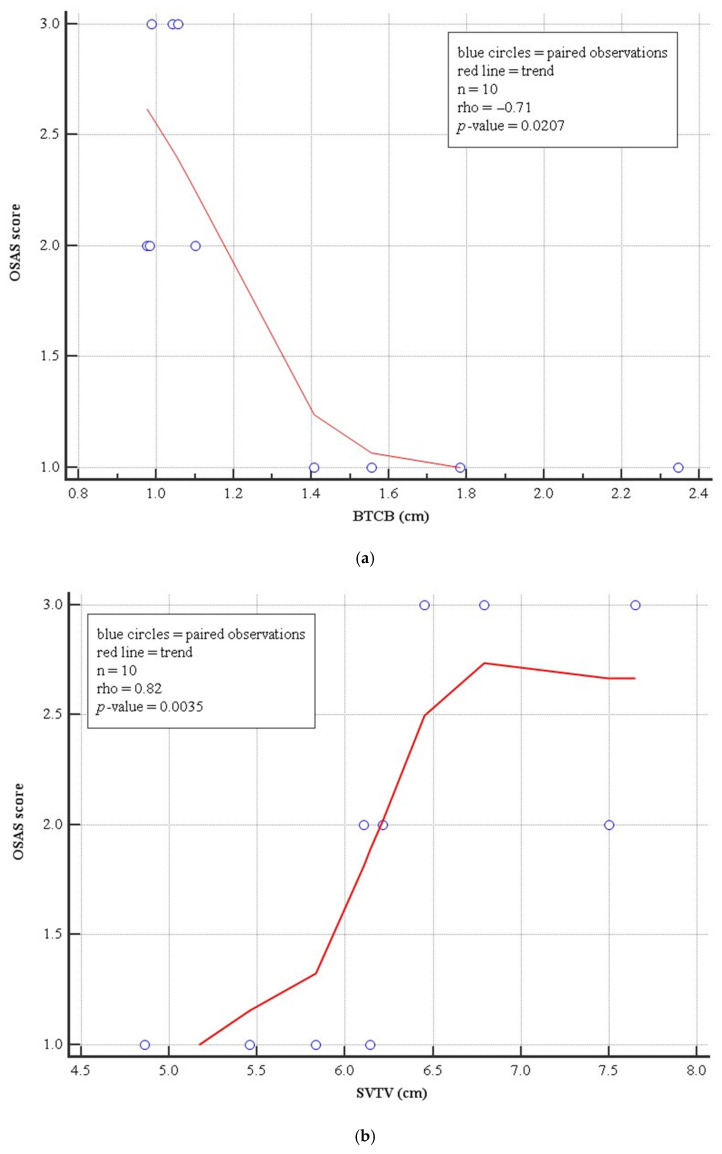
Significant relationships between OSA with BTCB (**a**) and SVTV (**b**).

Table 3 and Figure 2 showed a positive relationship between OSA degree and SVTV, while a negative relationship was observed between OSA and BTCB. In other words, an increase in the distance between the second cervical vertebra and the glottic plane, or a reduction in the distance between the base of the tongue and the vertebral body, increases the severity of obstructive sleep apnea.

For sleep quality parameters, a positive correlation between OSA and AHI was observed (0.94, *p* < 0.0001), i.e., an increase in apneas, hypopneas, or both increases the severity of obstructive sleep apnea.

Finally, no significant relationships were observed between OSA and global score of self-assessment questionnaires such as Stop-Bang, Epworth and Berlin.

## 4. Discussion

Generally, the anatomical subsites responsible for OSA are identified in the velum and at the level of the tongue and pharynx [23]. The only subsite considered at the laryngeal level is the epiglottis. In fact, some authors have highlighted, especially in patients intolerant to CPAP therapy, the presence of a double site of obstruction, with the epiglottis involved alone or in combination in approximately 30% of the cases considered [24,25].

There are sporadic publications in the literature identifying concomitant laryngeal pathologies as causes of OSA, such as vocal cord paralysis [26], cysts of the epiglottis [25] or false vocal cords [24], cysts of the aryepiglottic fold [25], laryngocele [25], and vocal cord prolapse [27].

Regarding the position of the larynx, it appears that the lower the larynx, or the greater the distance between the cricoid cartilage and the hard palate, the greater the risk of developing OSA [9,25,28]. It is also known that radiotherapy to the head and neck region causes alterations in the mechanoreceptors in the hypopharyngeal region, compromising the dilatory function of the hypopharynx [29].

Partial surgeries aim to achieve radical oncological treatment while ensuring functional recovery through the natural pathways of swallowing, breathing, and speaking with removal of the tracheotomy [15,17].

We will not discuss the indications and surgical technique, as they are not the subject of this paper.

The surgical procedure, in short, involves the removal of the entire thyroid cartilage along with the true vocal cords and the paralaryngeal spaces. The reconstructive procedure involves creating a space between the hyoid bone, the cricoid cartilage (CHP), and sometimes the suprahyoid epiglottis (CHEP) [4,29].

Specifically, in supracricoid laryngectomies, several anatomical factors are present that may be associated with an increased risk of OSA.

In particular, these include:

- the position of the base of the tongue, which is involved in the pexia and is pushed behind the hyoid bone and fixed to it;
- the laryngeal structures, lacking the support of the thyroid cartilage skeleton, which lose structural consistency, especially at the level of the valleculae;
- the arytenoids, which may be edematous and increase in volume, as may the neolarynx mucosa at the level of the pexia;
- the epiglottis, which may prolapse posteriorly when the sutures fixing it to the hyoid bone and the base of the tongue are not placed effectively [17,18,20,30].

Conversely, the diameter of the cricoid cartilage remains constant [4,7,9].

There is some debate in the literature regarding the onset of OSA during reconstructive laryngectomy and especially about the difference between horizontal and vertical reconstructive surgery, but the position of the larynx in relation to OSA has never been examined which, in patients undergoing supracricoid surgery, appears to be more cranial and not lower [4,7,9,18,21,24,31].

In our study, four patients presented mild OSA, three patients presented moderate OSA, and three patients severe OSA. Seven patients had a greater number of hypopnea episodes than apnea episodes; three patients had a greater number of apnea episodes than hypopnea episodes (Table 1).

The lack of a statistically significant correlation between the assessment questionnaires administered to patients and the AHI makes our work even more interesting. Based on a simple questionnaire, we risk underestimating OSA patients.

We excluded patients undergoing radiation therapy because an increased incidence of OSA (41%) has already been demonstrated in the literature [29].

The cause of this increased incidence is likely due to an alteration of the feedback between the laryngeal mechanoreceptors present in the mucosa and the dilator muscles in the pharynx, which is altered after radiation therapy [25].

Regarding the parameters considered, the Mallampati and Friedman classifications are consistent both before and after surgery.

The risk factors considered, such as smoking and BMI, were reduced in some cases after surgery. Particularly, in our cohort, the mean BMI was 28.1 ± 4.8 kg/m^2^, with most patients in the overweight range and only three patients classified as obese. To assess whether BMI acted as a confounding factor, we examined its relationship with OSA. The correlation between BMI and clinical OSA was not significant (Spearman’s rho = −0.07; *p* = 0.86). Likewise, no significant association was found between BMI and AHI considered as a continuous variable (Spearman’s rho = 0.12; *p* = 0.75). These findings indicate that, within our limited pilot sample, BMI did not predict OSA severity.

Importantly, all patients were evaluated at least one year after surgery, to minimize potential bias related to postoperative edema of the involved laryngeal structures. This reduces the likelihood that transient postoperative factors (e.g., edema, temporary functional adaptations) influenced the results and strengthened the relevance of the anatomical correlations observed and detected on CT scans in the evaluation of the anatomical changes that occurred after surgery and the onset of OSA. These parameters taken into consideration have already been evaluated in other studies with significant results [28,32,33].

In contrast to BMI, specific postoperative anatomical parameters (SVTV and BTCB) showed significant correlations with OSA, suggesting that structural changes following OPHL II may contribute independently to sleep-disordered breathing. In particular, the increase in the distance between the second cervical body and the glottic plane, and the reduction in the tongue–to–cervical-body distance (BTCB), which indicates the distance between the base of the tongue and the cervical body, are associated with an increased incidence of OSA in patients undergoing supracricoid surgery. These parameters are certainly influenced and undergo variations resulting from the surgical technique, particularly a reduction in retrolingual space. There is no relationship between the SVTH parameter and OSA, and this is very important because this measured distance does not vary after surgery.

Particularly after the thyroid cartilage removal, the anastomosis between the cricoid cartilage and the hyoid bone causes a downward pull on the latter, causing retraction of the tongue. The reduction in the anteroposterior retrolingual space correlates with increased OSA severity [9].

No assessments (polysomnography, questionnaires) were performed before surgery for two reasons: (1) oncological priority; (2) a tumor in the laryngeal region certainly represents an obstructive site that could lead to sleep-related breathing disorders.

### Limitations and Strengths of the Study

This study has several limitations that should be acknowledged. First, the sample size was small, as expected for a prospective pilot study, and was estimated using a wide margin of error due to the complete absence of previous data on the relationship between postoperative radiological parameters and OSA after type II OPHL. Although this represents a methodological limitation, the prospective design and the homogeneous surgical cohort strengthen the internal consistency of the results, which should nonetheless be interpreted with caution given the exploratory nature of the study. Second, a fully controlled prospective design with systematic preoperative polysomnography (PSG) would have been ideal; however, such an approach was not feasible in this oncologic surgical setting for ethical and logistical reasons. The lack of preoperative polysomnography does not allow differentiation between pre-existing and postoperative obstructive sleep apnea (OSA). Consequently, the findings should be interpreted as reflecting postoperative prevalence and associations, rather than implying a causal relationship. Therefore, patients were consecutively enrolled and evaluated after a long postoperative interval of at least one year, ensuring that the functional outcomes reflected stable, long-term conditions rather than transient postoperative changes.

Additional methodological considerations should also be noted. All patients were treated in a single Italian center and therefore represent a geographically homogeneous cohort. While this homogeneity may limit broader population generalizability, it also reduces variability and enhances internal consistency, which is advantageous in a pilot study aimed at exploring the anatomical and functional consequences of a standardized surgical procedure such as OPHL type II. Although the use of Spearman’s rank correlation coefficient helped mitigate some analytical bias by reducing the influence of non-normal distributions, outliers, and monotonic but non-linear relationships, residual confounding cannot be excluded. Furthermore, as in all observational studies, the possibility of measurement bias or under-/over-reporting of certain behaviors cannot be fully ruled out. Finally, since this is an exploratory study, the results will need to be confirmed in larger multicenter cohorts with standardized pre- and postoperative assessments. Despite these limitations, the study provides novel insights into the potential impact of postoperative anatomical changes on OSA, offering a valuable foundation for future research in an area where evidence is currently lacking.

This study presents several strengths that support its novelty and relevance. It is, to our knowledge, the first prospective investigation specifically addressing the relationship between postoperative anatomical changes following OPHL type II and the development of OSA, filling a complete gap in the current literature. A major strength lies in the integration of postoperative radiological measurements with long-term functional outcomes, offering a quantitative and reproducible assessment of airway remodeling in a surgical context traditionally described only qualitatively. The inclusion of a homogeneous cohort treated with the same surgical technique, combined with a minimum one-year follow-up, allowed the evaluation of stable functional adaptations rather than early postoperative fluctuations. The prospective design and systematic acquisition of radiological parameters further enhance methodological rigor. Overall, this study is pioneering because it introduces a quantitative framework for understanding how OPHL type II may influence OSA risk, establishing a foundation for future multicenter research in an area where evidence is currently lacking.

## 5. Conclusions

Based on the above findings, we believe there is a relationship between surgery and the onset of OSA. For this reason, multidisciplinary collaboration between an otolaryngologist, pulmonologist, and radiologist is essential for early diagnosis of OSA patients. This reduces the risk to the patient’s overall well-being as well as potentially reducing healthcare burden. Early treatment of an OSA patient reduces the long-term complications that this condition can cause.

It is necessary to expand the sample size further, perhaps by conducting a multicenter study to collect more data on changes in radiologically measured distances, to be able to propose a classification of the radiological laryngeal subsites responsible for the onset of OSA.

## Figures and Tables

**Table 1:** General characteristics of our sample.

Parameters	
*Patients*	10
*Age*	
Mean ± SD	64.8 ± 6.6
Median (IQR)	64.0 (60.0, 69.0)
*Gender*	
Males	80.0% (8)
Females	20.0% (2)
*OPHL II*	
Type A	60.0% (6)
Type B	40.0% (4)
*Smoke*	
Ex-smoker	90.0% (9)
Non-smoker	10.0% (1)
*Cigarette packs/year* (including Ex-smoker only)	
Mean ± SD	62.0 ± 20.3
Median (IQR)	60.0 (47.5, 78.5)
*BMI*	
Mean ± SD	28.1 ± 4.8
Median (IQR)	27.3 (25.4, 31.1)
*BMI category*	
Underweight	0.0% (0)
Normal weight	10.0% (1)
Overweight	60.0% (6)
Obese	30.0% (3)
*Mallampati*	
Class I	0.0% (0)
Class II	40.0% (4)
Class III	60.0% (6)
*Friedman*	
Degree I	0.0% (0)
Degree IIA	30.0% (3)
Degree IIB	20.0% (2)
Degree III	50.0% (5)
Degree IV	0.0% (0)
*Hypertension*	60.0% (6)
*Dyslipidemia*	70.0% (7)
*Type 2 diabetes mellitus*	10.0% (1)
*Cardiovascular disease*	10.0% (1)
*Nr hypopneas*	
Mean ± SD	33.7 ± 28.4
Median (IQR)	26.0 (12.75, 52.75)
*Nr. apneas*	
Mean ± SD	103.6 ± 107.1
Median (IQR)	58.5 (38.5, 138.0)
*Nr. Apneas/Nr hypopneas*	
Nr Apneas > Nr hypopneas	70.0% (7)
Nr Apneas = Nr hypopneas	0.0% (0)
Nr Apneas < Nr hypopneas	30.0% (3)
*Central apneas*	
Mean ± SD	4.4 ± 7.2
Median (IQR)	1.0 (0.25, 3.0)
*Mixed apneas*	
Mean ± SD	1.9 ± 3.3
Median (IQR)	0.0 (0.0, 2.5)

**Table 2:** Results by self-assessment questionnaires, sleep quality parameters, and radiological parameters after laryngectomy.

**OSA Severity**	
*OSA*	
Mild	40.0% (4)
Moderate	30.0% (3)
Severe	30.0% (3)
**Self-assessment questionnaires**	
*Stop-Bang*	
Mean ± SD	4.5 ± 1.8
Median (IQR)	4.5 (3.0, 6.0)
*Epworth*	
Mean ± SD	8.6 ± 3.5
Median (IQR)	8.5 (6.0, 10.0)
*Berlin*	
Low	50.0% (5)
High	50.0% (5)
**Sleep quality parameters**	
*AHI*	
Mean ± SD	25.5 ± 18.9
Median (IQR)	18.5 (13.1, 33.2)
*AHI degree*	
Mild ([5, 15[)	40.0% (4)
Moderate ([15, 30[)	30.0% (3)
Severe (≥30)	30.0% (3)
*ODI*	
Mean ± SD	23.5 ± 12.7
Median (IQR)	22.4 (13.0, 26.8)
*T90%*	
Mean ± SD	8.9 ± 8.1
Median (IQR)	9.0 (1.2, 14.0)
*T90% degree*	
Mild ([0, 5[)	40.0% (4)
Moderate ([5, 25[)	60.0% (6)
Severe (≥25)	0.0% (0)
*N90%*	
Mean ± SD	89.3 ± 2.2
Median (IQR)	90.0 (87.0, 91.0)
**Radiological parameters**	
*ALVC (cm)*	
Mean ± SD	6.721 ± 0.90
Median (IQR)	6.672 (5.934, 7.499)
*BTCB (cm)*	
Mean ± SD	1.324 ± 0.456
Median (IQR)	1.079 (0.988, 1.556)
*SVTH (cm)*	
Mean ± SD	7.963 ± 0.649
Median (IQR)	8.12 (7.585, 8.14)
*SVTV (cm)*	
Mean ± SD	6.30 ± 0.856
Median (IQR)	6.18 (5.832, 6.788)

**Table 3:** Correlations analysis between OSA and sleep quality and radiological parameters.

Correlation Analysis	rho (*p*-Value)	*p*-Value (Test)
**Sleep quality parameters**		
OSA/AHI	0.94 (<0.0001) *	
OSA/ODI	0.44 (0.20)	
OSA/T90%	0.11 (0.76)	
OSA/N90%	−0.14 (0.70)	
**Self-assessment questionnaire**		
OSA/Stop-Bang	−0.18 (0.62)	
OSA/Epworth	0.09 (0.80)	
OSA/Berlin		0.45 (F)
**Radiological parameters**		
OSA/ALVC	0.37 (0.30)	
OSA/BTCB	−0.71 (0.0207) *	
OSA/SVTH	−0.17 (0.63)	
OSA/SVTV	0.82 (0.0035) *	

* = significant test, rho = Spearman correlation coefficient, F = Generalized Fisher’s exact test.

## Data Availability

The data presented in this study are available on request from the corresponding author due to privacy and ethical considerations related to sensitive personal information.
