# Correction: Mesolella et al. Obstructive Sleep Apnea After Supracricoid Laryngeal Surgery (OPHL II): A Monocentric Prospective Pilot Study. *Cancers* 2026, *18*, 1212

**DOI:** 10.3390/cancers18132066

**Published:** 2026-06-25

**Authors:** Massimo Mesolella, Salvatore Allosso, Fabio Perrotta, Carlo Iadevaia, Carmela Cirillo, Nicola Serra, Pasquale Capriglione, Martina Ricciardiello, Anna Leoni, Anna Rita Fetoni

**Affiliations:** 1 Unit of Otorhinolaryngology, Department of Neuroscience, Reproductive Sciences and Dentistry, University of Naples Federico II, 80131 Naples, Italy; massimo.mesolella@unina.it (M.M.); pasquale.capriglione@unina.it (P.C.); martinaricciardielo@icloud.com (M.R.); annarita.fetoni@unina.it (A.R.F.); 2 Department of Translational Medical Sciences, University of Campania “L. Vanvitelli”, 80131 Naples, Italy; fabio.perrotta@unicampania.it (F.P.); dott.carlo.iadevaia@gmail.com (C.I.); carmela.cirillo1990@gmail.com (C.C.); leonianna4@gmail.com (A.L.); 3 Unit of Respiratory Medicine “Luigi Vanvitelli”, A.O. dei Colli, Monaldi Hospital, 80131 Naples, Italy; 4 Department of Neuroscience, Reproductive Sciences and Dentistry, Audiology Section, University of Naples Federico II, Via Pansini 5, 80131 Naples, Italy; 5 Hearing and Balance Unit, Department of Head and Neck, Federico II University Hospital, Via Pansini 5, 80131 Naples, Italy

Addition of a Corresponding Author

Dr. Nicola Serra was inadvertently not included as a corresponding author in the original publication due to a typographical error in manuscript preparation [1]. The authors state that the scientific conclusions are unaffected. This correction was approved by the Academic Editor. The original publication has also been updated.
